# Assessment of Quality of Life in Lithuanian Patients with Multimorbidity Using the EQ-5D-5L Questionnaire

**DOI:** 10.3390/medicina61020292

**Published:** 2025-02-08

**Authors:** Olga Vasiliauskienė, Dovydas Vasiliauskas, Aušrinė Kontrimienė, Lina Jaruševičienė, Ida Liseckienė

**Affiliations:** 1 Department of Family Medicine, Lithuanian University of Health Sciences, LT-44307 Kaunas, Lithuania; 2 Department of Chemistry, The University of Chicago, Chicago, IL 60637, USA; 3 Department of Public Health, Lithuanian University of Health Sciences, LT-44307 Kaunas, Lithuania

**Keywords:** EQ-5D-5L, SF-36, quality of life, multimorbidity, chronic disease, primary healthcare

## Abstract

*Background and Objectives*: Despite the critical importance of effective healthcare management for patients with multimorbidity, robust and reliable tools for assessing health-related quality of life in Lithuania remain scarce. We aim to identify trends in the quality of life of patients with multimorbidity and to evaluate the effectiveness of the Lithuanian version of the EuroQol EQ-5D-5L questionnaire. *Materials and Methods*: The study included patients between the ages of 40 and 85 (N = 498) who had at least two chronic conditions, arterial hypertension being a prerequisite. The participants completed a comprehensive set of questionnaires specifically prepared for the TELELISPA “Improved healthcare quality for patients with multimorbidity in Lithuania” project which included the translated EQ-5D-5L questionnaire. The predictive validity of the EQ-5D-5L questionnaire was assessed using correlations with the SF-36 and EQ-VAS scores, a random forest regression model. Reliability was evaluated using Cronbach’s alpha and inter-item correlations. Trends in the quality of life in different patient groups were assessed with Chi-square tests. *Results*: The EQ-5D-5L questionnaire demonstrated high reliability and validity with a Cronbach’s alpha value of 0.737, EQ-5D-5L random forest machine learning regression model RMSE value of 0.1396, and adequate scores from other measures. Lower quality of life was found in patients with multimorbidity who had chronic conditions such as angina pectoris, heart failure, atrial fibrillation, or joint diseases, as well as the patients who were older than 60 years of age, women, or unemployed. Different aspects of quality of life were also significantly negatively impacted by diabetes, asthma, and chronic kidney disease. Heart failure, joint diseases, and older age had the biggest negative effect on quality of life. *Conclusions*: It is found that the Lithuanian EQ-5D-5L questionnaire is suitable for the assessment of the quality of life in patients with multimorbidity and indicates lower quality of life among those with specific cardiovascular and joint disorder chronic conditions and, in particular, demographic groups.

## 1. Introduction

Multimorbidity is becoming one of the most important public healthcare issues in aging populations around the world and Lithuania where more than half of the 65–75-year-old patients have two or more chronic disorders [1,2,3]. Providing satisfactory healthcare for patients with multimorbidity requires more availability of visits to primary healthcare facilities, more appointments with the members of primary healthcare teams and medical specialists, more frequent in-home physician visits, and more frequent and longer hospitalizations [2,4,5]. Altogether, such increased demand for healthcare resources places an additional burden on the healthcare systems and increases medical costs [6].

Studies indicate that patients with multimorbidity tend to experience lower quality of life and its associated problems [7]. The decline in the quality of life in patients with multimorbidity has been shown to correlate with various physical issues such as reduced mobility, chronic pain, fatigue, and discomfort [8]; psychological issues such as stress, anxiety, and depression [9]; and social/socioeconomic issues such as isolation [10] and increased treatment burden [11]. Learning and addressing the multi-faceted problems that negatively affect the quality of patients’ lives requires the development and application of life quality evaluation tools. Certain tools such as the Medical Outcomes Study Short Form Health Survey SF-36, its shorter counterpart SF-12, the EuroQol 5-Dimension 5-Level EQ-5D-5L, and the 3-Level EQ-5D-3L quality-of-life questionnaires have seen growing successful global application over the past years [12,13,14,15,16]. The greatest strengths of the EQ-5D questionnaires are their simplicity and ease of use, as well as the comprehensive coverage of the most important patient-affecting problems—their ability to move, engage in day-to-day activities, and practice self-care; the level of pain/discomfort; and the level of anxiety/depression [14,15,16]. Some other advantages of the EQ-5D questionnaires are their patient-centered nature, their versatility, applicability for different settings (such as population health surveys, clinical trials, and healthcare outcome measurements), and transferability between the different versions of the questionnaires released worldwide [14,15,16].

In this study, we aim to explore the quality of life of patients with multimorbidity in Lithuania and assess the validity and reliability of the Lithuanian version of the EQ-5D-5L questionnaire. The insights gained from this study are expected to enhance our understanding and contribute to the development of more nuanced, patient-centered healthcare strategies for multimorbid patients in Lithuania.

## 2. Materials and Methods

### 2.1. Study Design

The study was conducted in five urban-based and two rural-based Lithuanian primary healthcare centers in the Kaunas, Šiauliai, and Tauragė region municipalities between August and October 2021 as a part of TELELISPA project “Improved healthcare quality for patients with multimorbidity in Lithuania” (project no. 08.4.2-ESFA-K-616-01-0003). The study included respondents who were 40–85 years of age (N = 514) and had two or more chronic diseases (one of which had to be arterial hypertension).

### 2.2. Data Collection

The participants completed a comprehensive set of questionnaires specifically prepared for the project TELELISPA which included the translated EQ-5D-5L questionnaire, the SF-36 questionnaire, sociodemographic data tables, and the EQ-VAS health condition scale (EQ visual analog scale—a 20 cm length scale ranging from 0 to 100 where 0 is the worst imaginable health state and 100 is the best imaginable health state) [17]. The translated and pilot-tested Lithuanian version of the EQ-5D-5L questionnaire was received from the EuroQoL group (link provided in the Supplementary Materials). The face validity of the questionnaire was qualitatively evaluated and deemed acceptable by the TELELISPA study expert team.

Each of the EQ-5D-5L questionnaire dimensions (mobility, self-care, usual activities, pain/discomfort, and anxiety/depression) was divided into five strata (1—no associated problems, 2—slight problems, 3—moderate problems, 4—severe problems, and 5—extreme problems/unable to carry out the activity). Because the Lithuanian value set for EQ-5D-5L has not been released as of yet, the categorical EQ-5D-5L value sets were converted into the continuous utility value scale using the Polish value set that has been deemed to be suitable for Central and Eastern European countries lacking their own value sets [18]. The obtained utility value set had a minimum of −0.59 (corresponding to the categorical value of ‘55555’—the worst state for each of the questionnaire strata) and a maximum of 1.0 (corresponding to the categorical value of ‘11111’—the best state for each of the questionnaire strata).

### 2.3. Statistical Analysis

The Chi-square (χ^2^) was used to compare the proportions of study subjects with different chronic conditions (certain chronic cardiovascular, respiratory, endocrine, kidney, and joint diseases) and from different sociodemographic groups (age, sex, education, occupation, and residential area) who reported the best EQ-5D-5L health state “11111” and other health states. The same Chi-square (χ^2^) analysis was used to compare the proportions of the best health state and other health states in each of the EQ-5D-5L categories (mobility, self-care, usual activities, pain/discomfort, and anxiety/depression). More details about the group comparisons are provided in the footnotes of the result tables.

The internal consistency reliability of the questionnaire was determined with Cronbach‘s alpha value (estimated ideal reliability interval 0.7–0.9) [19] and the inter-item correlations (ideal reliability interval 0.15–0.50) [20]. The validity of the questionnaire was determined by examining the correlations between the EQ-5D-5L utility value scale and the average scores of the EQ-VAS scale and different categories of the SF-36 questionnaire. Validity was also verified with the random forest regression machine learning model. The random forest model predicts regression interval values by creating many decision trees based on collections of random learning parameters and averaging out the predictions from separate trees (Supplementary Materials—Figure S1) [21]. The model included age; sex; education; occupation; the presence/absence of chronic cardiovascular, joint, lung, and kidney diseases; and the score of the SF-36 questionnaire item one (“In general, would you say your health is:”) as features. It utilized 100 decision trees and 70/30 percent of study participants in training/testing groups, respectively. The regression prediction accuracy for the training and testing samples was determined with the root mean square error (RMSE) measure. The value of the 95th percentile in the training error distribution was used as the threshold for the evaluation of the training/testing error distribution similarity.

The relative informativity power of the EQ-5D-5L questionnaire and the evenness of answer frequencies across strata 1–5 were evaluated with the Shannon index (entropy) $H^{\prime }$ and Shannon's evenness index $J^{\prime }$ [22]. The Shannon index is defined as

(1) $$H^{\prime }=-\sum_{i=1}^{L}p_{i}{log}_{2}p_{i}$$

where L signifies the number of available strata (in our case, five), and $p_{i}=n_{i}/N$ signifies the proportion of responses in each of the five strata i. Due to a fixed entropy maximum (2.322 for our five-strata case), response distribution across the strata can be objectively evaluated with the Shannon evenness index $J^{\prime }$ which is defined as

(2) $$J^{\prime }=\frac{H\prime }{{H\prime }_{max}}$$

where ${H\prime }_{max}$ is the entropy maximum. In addition, the ceiling effect was calculated as the proportion of respondents who indicated they had “no problems” with the activity specified in any of the five questionnaire categories.

## 3. Results

### 3.1. Sociodemographic Data

Out of the entire study population (N = 514), 498 (96.8%) of the subjects responded to all the EQ-5D-5L questionnaire items. About one-third of the respondents were younger than 60 years (29.1%) or older than 70 years (30.1%) of age. Women made up a small majority with 59.8% of the study population. More than two-thirds of the participants had university or professional education (68.9%), about one-third of the participants (34.9%) were employed, and almost half (44.6%) had retired. Half of the participants (49.8%) reported they had four or more chronic disorders, 41.5% had cardiovascular disorders other than arterial hypertension, and about one-third had heart failure (33.7%) and diabetes (35.3%). Details on the additional sociodemographic data are provided in Table 1.

### 3.2. EQ-5D-5L Data Overview

The EQ-5D-5L questionnaire had an overall ceiling effect of 16%. More than half of the respondents indicated they did not have problems with either self-care (81.7%), usual activities (65.4%), or anxiety/depression (57.6%) (Table 2). Almost half of the respondents reported no problems with mobility (48.8%) while only a quarter of the respondents (25.3%) had no problems with pain/discomfort. The only categories that had more than 5% of the study participants report severe or extreme problems were pain/discomfort (7.6%) and mobility (5.2%). The Shannon index and Shannon evenness index (Supplementary Materials—Table S1) showed that the responses were most evenly distributed in the pain and mobility categories (with respective J′ values of 0.791 and 0.731), whereas the lowest evenness was observed in the self-care category ($J^{\prime }$ = 0.379).

### 3.3. EQ-5D-5L: Sociodemographic Correlations

The Chi-square test analysis indicated that the patients with multimorbidity who were younger than 60 years of age had significantly better EQ-5D-5L scores than older patients (*p* = 0.001) (Table 3). The men reported a higher quality of life than the women (*p* = 0.01), whereas the working patients reported they had a better quality of life than the non-working and retired patients. There were also multiple category-specific differences across the study participant groups. Significantly lower difficulty with mobility was reported by patients younger than 60 years of age (*p* = 0.03), patients with a university education (*p* = 0.01), and employed patients (*p* < 0.001). The employed patients (*p* < 0.001) and patients with a university education (*p* = 0.02) reported they experienced less difficulty with usual activities. The men (*p* < 0.001), respondents with a university education (*p* = 0.04), and employed patients (*p* = 0.03) suffered less from pain-related problems. Lesser anxiety- and depression-related problems were indicated by the men (*p* = 0.002) and patients who were older than 60 years of age (*p* = 0.007). The working patients reported significantly fewer problems with self-care (*p* < 0.001).

### 3.4. EQ-5D-5L: Chronic Disease Correlations

Statistically significant differences were observed between certain EQ-5D-5L categories and chronic diseases (Table 4). Significantly worse quality of life was reported by the patients who had angina pectoris (*p* = 0.025), heart failure (*p* = 0.026), and atrial fibrillation (*p* = 0.016). Poorer mobility was experienced by the patients with cardiovascular diseases (*p* = 0.040), heart failure (*p* < 0.001), atrial fibrillation (*p* = 0.004), and joint diseases (*p* = 0.004). More significant obstacles in self-care were reported by the respondents with heart failure (*p* = 0.006) and joint diseases (*p* = 0.002). Increased difficulty in engaging with daily activities was reported by the patients with heart failure (*p* < 0.001), asthma or COPD *p* = 0.005), joint diseases (*p* = 0.003), osteoporosis (*p* = 0.011), and chronic kidney disease (*p* = 0.016). The respondents indicated greater problems with pain if they had diabetes (*p* = 0.013) and joint diseases (*p* = 0.035). Higher levels of anxiety/depression were reported by the patients who had heart failure (*p* < 0.001), atrial fibrillation (*p* = 0.035), asthma (*p* = 0.002), and osteoporosis (*p* = 0.001).

### 3.5. Reliability and Validity Analysis

The Cronbach’s alpha value of the EQ-5D-5L questionnaire (0.737) fell into the ideal reliability interval (0.7–0.9). All the inter-item correlations fell into the ideal interval of 0.15–0.50 or minimally exceeded 0.50 (Supplementary Materials—Table S2). Correlations between the EQ-5D-5L utility value sets and SF-36 question groups as well as the EQ VAS values were all statistically significantly higher than zero (Table 5). Moderately strong correlations were found between the EQ-5D-5L index values and SF-36 item one values (r = 0.479), EQ VAS values (r = 0.401), SF-36 physical functioning item values (r = 0.576), SF-36 energy/fatigue item group (r = 0.423), SF-36 pain group (r = 0.541), and the SF-36 general health group (r = 0.403). Moderately strong negative correlations were found between the values in separate EQ-5D-5L categories and the other measures. The separate EQ-5D-5L categories had moderate correlations ranging from −0.648 to −0.550, and moderate-to-weak correlations with EQ VAS values that ranged between −0.381 and −0.179. The strongest correlations with the values of other measures were found for the pain, mobility, and usual activity categories while the weakest correlations were observed for the anxiety/depression category.

More information about the validity of the questionnaire was obtained from the random forest machine learning method. The RMSE was found to be 0.0565 for the training set and 0.1396 for the testing set (Supplementary Materials—Table S3). The 95th percentile of the training dataset RMSE distribution (0.116) was larger than 65.71% of the values in the validation set (Supplementary Materials Figure S2).

## 4. Discussion

The results of the study indicate a number of significant relationships between the EQ-5D-5L quality of life scores, membership in particular demographic groups, and the presence of specific chronic disorders in patients with multimorbidity. The EQ-5D-5L questionnaire is also found to have strong validity and reliability scores which suggest it is suitable for assessing the quality of life in patients with multimorbidity in Lithuania.

### 4.1. Questionnaire Validity and Reliability Analysis

The satisfactory reliability of the questionnaire is indicated by the good Cronbach’s alpha value (0.737) and appropriate inter-item correlations (0.15–0.50). The validity of the questionnaire is supported by statistically significant correlations between the EQ-5D-5L values and other patient health measures such as the scores of the SF-36 questionnaire and the EQ visual analog scale. Moderately high correlations between the questionnaire score and most of the SF-36 item groups (physical functioning, energy/fatigue, pain, and general health; r > 0.40; *p* < 0.001) suggest that the overall EQ-5D-5L score accurately reflects the effect of different aspects of health on the patients’ quality of life. The statistically significant correlations between the EQ VAS scores and most of the EQ-5D-5L and SF-36 groups (Table 5) suggest that the EQ VAS scale is a good predictor of the scores in certain EQ-5D-5L categories (mobility, self-care, etc.). The validity of the questionnaire is also supported by the random forest machine learning model results. The obtained RMSE regression value for the validation set (0.1396) was comparable to EQ-5D-5L utility scale RMSE values reported in earlier studies [23].

The good validity and reliability of the questionnaire are also demonstrated by Shannon entropy index values which were higher than 1.0 for all the EQ-5D-5L categories except for the self-care category. The moderately broad response variety indicates that the study participants did not have problems distinguishing and understanding what was asked by the different questionnaire categories while each questionnaire category had its distinctive effect on quality of life. The overall ceiling effect of the questionnaire and its separate categories ranged between 25 and 65%, similar to the previously published EQ-5D-5L validity studies [23,24,25]. The lowest ceiling effect (25.3%) was observed in the pain category, which indicates that the study participants frequently experience at least some degree of pain or discomfort. It is important to note that similarly to certain previously introduced foreign EQ-5D-5L questionnaires, the Lithuanian version of the questionnaire had a lower Shannon entropy value (=0.880) [23,26] in the self-care EQ-5D-5L category than the established satisfactory entropy threshold of 1.0. This may imply that both Lithuanian and foreign patients are not sufficiently informed about areas that are encompassed by the concept of self-care and may disregard the difficulties that are related to them.

### 4.2. Quality of Life in Sociodemographic and Chronic Disease Data Groups

The EQ-5D-5L questionnaire scores of patients with multimorbidity had statistically significant, logical relationships with the incidence of certain chronic disorders. Chi-square analysis shows that patients experienced lower quality of life if they had any cardiovascular diseases such as heart failure, atrial fibrillation, and angina pectoris, all of which have been shown to negatively affect quality of life [27,28,29,30]. Heart failure and joint diseases had the biggest negative effect on patients’ quality of life and were each associated with increased problems in at least four of the EQ-5D-5L questionnaire categories. Significant previously reported relationships were observed between the presence of chronic kidney disease and poorer engagement in usual activities [31], asthma and anxiety/depression, and diabetes and pain [32].

There were also notable differences in the questionnaire values among groups of certain sociodemographic categories. In accordance with previous studies [7,8,33,34], the patients with multimorbidity who were younger than 60 years of age reported better overall quality of life scores, and fewer problems with mobility, self-care, and anxiety/depression than the older patients. The women respondents reported worse quality of life and more pronounced problems with anxiety/depression than the men [35]. The patients with a university education were found to have fewer problems with mobility, usual activities, and pain than patients without a university education. The working patients with multimorbidity reported better overall quality of life and fewer problems in the majority of the questionnaire categories (except for the anxiety/depression category) in comparison to the unemployed/retired patients. Altogether, these statistically significant relationships that stand in agreement with observations from previous studies indicate that the Lithuanian version of EQ-5D-5L adequately reflects the quality of life of patients with multimorbidity.

### 4.3. Limitations and Future Directions

A potential limitation of the study is its relatively small sample size which may weaken the regression predictions in the random forest machine learning model. It should also be considered that some of the results might lose statistical significance after Bonferroni correction when multiple statistical tests are conducted for a single dataset. Furthermore, longitudinal studies could provide valuable insights into the temporal stability and predictive validity of the findings, thereby adding to the validity and applicability of the EQ-5D-5L questionnaire in different contexts.

One of the main advantages of adapting the EQ-5D-5L version is the opportunity to introduce new strategies for patients and healthcare teams in managing health-related trade-offs. Taken with other measures of patient health, the questionnaire has the potential to help create more supportive, effective, affordable patient-centered care [36] for patients with multimorbidity in Lithuania.

## 5. Conclusions

The Lithuanian version of the EQ-5D-5L questionnaire is demonstrated to have robust validity and reliability through various assessments, including the correlation with other life quality and health measures, the Shannon index, a random forest regression machine learning model, Cronbach’s alpha, and inter-item correlations. Consequently, this instrument effectively measures the quality of life in patients with multimorbidity. The study results reveal that lower quality of life is evident among Lithuanian patients with multimorbidity who have chronic diseases such as angina pectoris, heart failure, atrial fibrillation, and joint diseases or belong to specific sociodemographic groups, including individuals aged 60 and above, women, or those who are unemployed.

## Figures and Tables

**Table 1:** Participant characteristics.

	N = 498	%
Mean age (SD), years	65.1(9.4)	
Age years (missing data: *n* = 10, 2.0%)		
<60	142	29.1
60–64	107	21.9
65–70	92	18.9
70+	147	30.1
Sex (missing data: *n* = 0; 0%)		
Male	200	40.2
Female	298	59.8
Educational level (missing data: *n* = 5, 1.0%)		
Early education (0–10 years)	107	21.7
High school (11–12 years)	43	8.7
Some tertiary education (13–14 years)	183	37.1
University (14+ years)	157	31.8
Other	3	0.6
Employment status (missing data: *n* = 3, 0.6%)		
Employed	173	34.9
Unemployed	49	9.9
Employed retire	38	7.7
Retired	221	44.6
Other	14	2.8
Number of self-reported long-term conditions (IQR)	3.0	3.0–5.0
2	48	9.6
3	202	40.6
4	121	24.3
5+	127	25.5
Self-reported long-term conditions		
Hypertension	498	100.0
Cardiovascular Disease	208	41.5
Chronic Ischemic Heart Disease	54	10.8
Heart Failure	168	33.7
Atrial Fibrillation	84	16.9
Diabetes	176	35.3
Thyroid Gland Diseases	57	11.4
Hypothyroidism	12	2.4
COPD	20	4.0
Asthma	54	10.8
Joint Disease	19	3.8
Osteoporosis	16	3.2
Chronic kidney disease	31	6.2
Average health measure scores		
SF-36 question item 1 score (SD) ^a^		2.32 (0.70)
EQ VAS scale score (SD) ^b^		62.87 (16.6)

^a^ calculated based on the responses to SF-36 item 1 “In general, would you say your health is:” (1—excellent, 2—very good, 3—good, 4—fair, and 5—poor). ^b^ calculated based on the EQ VAS scale values (0–100).

**Table 2:** EQ-5D-5L questionnaire response frequency distribution for different questionnaire categories.

	Mobility, (%)	Self-Care, (%)	Usual Activities, (%)	Pain/Discomfort, (%)	Anxiety/Depression, (%)
1 (no problems)	243 (48.8)	407 (81.7)	326 (65.5)	126 (25.3)	287 (57.6)
2 (slight problems)	129 (25.9)	64 (12.9)	95 (19.1)	183 (36.7)	136 (27.3)
3 (moderate problems)	100 (20.1)	24 (4.8)	66 (13.3)	151 (30.3)	60 (12.0)
4 (severe problems)	26 (5.2)	2 (0.4)	9 (1.8)	38 (7.6)	13 (2.6)
5 (extreme problems/unable to carry out)	0 (0)	1 (0.2)	2 (0.4)	0 (0)	2 (0.4)

**Table 3:** EQ-5D-5L response distribution across different sociodemographic groups.

				*p*-Values ^a^	*p*-Values in Different EQ-5D-5L Groups ^b^				
	N	11111 State *, %	Other States *, %	EQ-5D-5L	Mobility	Self-Care	Usual Activities	Pain/Discomfort	Anxiety/Depression
Age groups									
<60	142	25.4	74.6	***p* = 0.001, χ^2^ (1) = 16.126**	***p* = 0.026, χ^2^ (1) = 18.946**	***p* = 0.0454, χ^2^ (1) = 11.898**	*p* = 0.182, χ^2^ (1) = 16.198	*p* = 0.233, χ^2^ (1) = 11.664	***p* = 0.007, χ^2^ (1) = 27.273**
60–64	107	15.9	84.1						
65–70	92	12.0	88.0						
70+	147	8.8	91.2						
Sex									
Male	200	21.0	79.0	***p* = 0.010, χ^2^ (1) = 6.607**	*p* = 0.097, χ^2^ (1) = 6.326	*p* = 0.371, χ^2^ (1) = 4.266	*p* = 0.190, χ^2^ (1) = 6.130	***p* < 0.001, χ^2^ (1) = 21.672**	***p* = 0.002, χ^2^ (1) = 16.849**
Female	298	12.4	87.6						
Education									
Elementary	107	15.9	84.1						
Secondary	43	16.3	83.7						
Professional	183	13.7	86.3						
University	157	17.8	82.2	*p* = 0.376, χ^2^ (1) = 0.784	***p* = 0.011, χ^2^ (1) = 25.846**	*p* = 0.076, χ^2^ (1) = 24.659	***p* = 0.020, χ^2^ (1) = 29.608**	***p* = 0.038, χ^2^ (1) = 21.989**	*p* = 0.297, χ^2^ (1) = 18.474
Other	3	0	100						
Employment									
Employed	173	26.6	73.4	***p* < 0.001, χ^2^ (1) = 24.649**	***p* < 0.001, χ^2^ (1) = 42.066**	***p* < 0.001, χ^2^ (1) = 46.020**	***p* < 0.001, χ^2^ (1) = 50.260**	***p* = 0.035, χ^2^ (1) = 22.275**	*p* = 0.485, χ^2^ (1) = 15.549
Unemployed	49	8.2	91.8						
Retired and working	38	18.4	81.6						
Retired	221	9.5	90.5						
Other	14	7.1	92.9						

* proportions of participants who chose the questionnaire option “1—no problems” for each of the questionnaire categories (11111 state) or any other option combinations (other states). ^a^ the Chi-square (χ^2^) test results for proportions of study participants who recorded the best imaginable state (11111) or any other states; for each of the sociodemographic groups, the category that has the listed significance values is compared to the other categories. Statistically significant differences are highlighted in bold. ^b^ the Chi-square (χ^2^) test results for proportions of study participants who selected the option “1—no problems” or any other options for separate EQ-5D-5L questionnaire categories. Statistically significant results at the α = 0.05 level are highlighted in bold. Note: Chi-square test values in parentheses—degrees of freedom.

**Table 4:** EQ-5D-5L response distribution among patients with different chronic disorders.

				*p*-Values ^a^	EQ-5D-5L *p*-Values for Separate Groups ^b^				
	Absent/Present	*n*	%	EQ-5D-5L	Mobility	Self-Care	Usual Activities	Pain/Discomfort	Anxiety/Depression
I20 Angina pectoris	A	290	58.23						
	P	208	41.77	***p* = 0.025, χ^2^ (1) = 5.006**	***p* = 0.040, χ^2^ (1) = 8.326**	*p* = 0.217, χ^2^ (1) = 5.769	*p* = 0.360, χ^2^ (1) = 4.356	*p* = 0.093, χ^2^ (1) = 6.410	*p* = 0.078, χ^2^ (1) = 8.409
I25 Chronic ischemic heart disease	A	444	89.16						
	P	54	10.84	*p* = 0.823, χ^2^ (1) = 0.050	*p* = 0.414, χ^2^(1) = 2.856	*p* = 0.122, χ^2^ (1) = 7.269	*p* = 0.364, χ^2^ (1) = 4.325	*p* = 0.399, χ^2^ (1) = 2.950	*p* = 0.446, χ^2^ (1) = 3.717
I50 Heart failure	A	330	66.27						
	P	168	33.73	***p* = 0.026, χ^2^ (1) = 5.036**	***p* < 0.001, χ^2^ (1) = 31.843**	***p* = 0.006, χ^2^ (1) = 14.515**	***p* < 0.001, χ^2^ (1) = 28.245**	*p* = 0.070, χ^2^ (1) = 7.059	***p* < 0.001, χ^2^ (1) = 20.504**
I48 Atrial fibrillation and flutter	A	414	83.13						
	P	84	16.87	***p* = 0.016, χ^2^ (1) = 5.757**	***p* = 0.004,** **χ^2^ (1) = 13.268**	*p* = 0.379, χ^2^ (1) = 4.207	*p* = 0.125, χ^2^ (1) = 7.214	*p* = 0.795, χ^2^ (1) = 1.026	***p* = 0.035, χ^2^ (1) = 10.317**
E11 Type II diabetes mellitus	A	322	64.66						
	P	176	35.34	*p* = 0.593, χ^2^ (1) = 0.285	*p* = 0.959, χ^2^ (1) = 0.307	*p* = 0.526, χ^2^ (1) = 3.194	*p* = 0.849, χ^2^ (1) = 1.371	***p* = 0.013 χ^2^ (1) = 10.810**	*p* = 0.121 χ^2^ (1) = 7.292
E06 Thyroiditis	A	441	88.55						
	P	57	11.45	*p* = 0.688, χ^2^ = 0.161	*p* = 0.929, χ^2^ = 0.452	*p* = 0.780, χ^2^ = 1.761	*p* = 0.845, χ^2^ = 1.396	*p* = 0.686, χ^2^ = 1.485	*p* = 0.610, χ^2^ = 2.695
E89 Postprocedural endocrine and metabolic disorders	A	486	97.59						
	P	12	2.41	*p* = 0470, χ^2^ (1) = 0.522	*p* = 0.758, χ^2^ (1) = 1.180	*p* = 0.709, χ^2^ (1) = 2.147	*p* = 0.710, χ^2^ (1) = 2.139	*p* = 0.599, χ^2^ (1) = 1.874	*p* = 0.594, χ^2^ (1) = 2.790
J44 COPD	A	478	95.98						
	P	20	4.02	*p* = 0.464, χ^2^ (1) = 0.537	*p* = 0.869, χ^2^ (1) = 0.718	*p* = 0.356, χ^2^ (1) = 4.392	***p* = 0.005, χ^2^ (1) = 14.739**	*p* = 0.464, χ^2^ (1) = 2.561	*p* = 0.602, χ^2^ (1) = 2.741
J45 Asthma	A	444	89.16						
	P	54	10.84	*p* = 0.823, χ^2^ (1) = 0.050	*p* = 0.179, χ^2^ (1) = 4.901	*p* = 0.980, χ^2^ (1) = 0.434	*p* = 0.794, χ^2^ (1) = 1.682	*p* = 0.794, χ^2^ (1) = 1.682	***p* = 0.002, χ^2^ (1) = 17.500**
M05/M06 Joint diseases	A	479	96.18						
	P	19	3.82	*p* = 0.054, χ^2^ (1) = 3.724	***p* = 0.007, χ^2^ (1) = 12.206**	***p* = 0.002, χ^2^ (1) = 17.159**	***p* = 0.003, χ^2^ (1) = 15.907**	***p* = 0.035, χ^2^ (1) = 8.614**	***p* = 0.006, χ^2^ (1) = 14.532**
M80/M81 Osteoporosis	A	482	96.79						
	P	16	3.21	*p* = 0.708, χ^2^ (1) = 0.140	*p* = 0.838, χ^2^ (1) = 0.847	*p* = 0.577, χ^2^ (1) = 2.886	***p* = 0.011, χ^2^ (1) = 13.025**	*p* = 0.396, χ^2^ (1) = 2.970	***p* = 0.001, χ^2^ (1) = 17.588**
N18 CKD	A	467	93.78						
	P	31	6.22	*p* = 0.330, χ^2^ (1) = 0.948	*p* = 0.705, χ^2^ (1) = 1.403	*p* = 0.721, χ^2^ (1) = 2.082	***p* = 0.016, χ^2^ (1) = 12.232**	*p* = 0.838, χ^2^ (1) = 0.847	*p* = 0.687, χ^2^ (1) = 2.265

^a^ the Chi-square (χ^2^) test results for proportions of study participants who recorded the best imaginable state (11111) or any other states; for each of the sociodemographic groups, the category that has the listed significance values is compared to the other categories. Statistically significant differences are highlighted in bold. ^b^ the Chi-square (χ^2^) test results for proportions of study participants who selected the option “1—no problems” or any other options for separate EQ-5D-5L questionnaire categories. Statistically significant results at the α = 0.05 level are highlighted in bold.

**Table 5:** Correlations between the EQ-5D-5L index values, SF-36 item groups, and EQ VAS scale values (95% CI—confidence interval).

	EQ-5D-5L Index		SF-36 HT ^&^		EQ VAS	
	Correlation	95% CI *	Correlation	95% CI	Correlation	95% CI
EQ-5D-5L index			0.456	(0.381, 0.526)	0.391	(0.314, 0.464)
SF-36 HT ^&^					0.462	(0.387, 0.531)
SF-36 PF ^&^	0.576	(0.512, 0.634)			0.408	(0.329, 0.481)
SF-36 PH ^&^	0.308	(0.221, 0.390)			0.217	(0.126, 0.303)
SF-36 EP ^&^	0.158	(0.066, 0.248)			0.057	(−0.036, 0.149)
SF-36 EF ^&^	0.423	(0.345, 0.496)			0.424	(0.346, 0.496)
SF-36 EWB ^&^	0.278	(0.191, 0.360)			0.313	(0.229, 0.393)
SF-36 SCF ^&^	0.356	(0.273, 0.433)			0.238	(0.150, 0.322)
SF-36 P ^&^	0.541	(0.473, 0.603)			0.361	(0.279, 0.438)
SF-36 GH ^&^	0.403	(0.324, 0.477)			0.484	(0.411, 0.551)
1 Mobility ^a^	−0.648	(−0.697, −0.594)	−0.388	(−0.464, −0.308)	−0.372	(−0.446, −0.293)
2 Self-care	−0.593	(−0.648, −0.533)	−0.333	(−0.412, −0.249)	−0.262	(−0.342, −0.178)
3 Usual activities	−0.638	(−0.687, −0.582)	−0.417	(−0.490, −0.339)	−0.381	(−0.454, −0.303)
4 Pain/discomfort	−0.820	(−0.847, −0.788)	−0.382	(−0.457, −0.301)	−0.301	(−0.380, −0.219)
5 Anxiety/depression	−0.550	(−0.609, −0.486)	−0.241	(−0.325, −0.153)	−0.179	(−0.263, −0.092)

Note: All the reported correlations are significantly higher or lower than zero. * 95% CI—confidence interval. ^a^ the numerical values for each of the five EQ-5D-5L categories (mobility, self-care, etc.) were expressed in strata 1–5 with respect to the selected answer options (1—no problems, 2—slight problems, etc.). ^&^ correlations for different SF-36 items/categories (HT—item one (“In general, would you say your health is…”); PF—physical functioning item group; PH—role limitations due to physical health group; EP—role limitations due to emotional problem group; EF—energy/fatigue group; EWB—emotional well-being group; SCF—social functioning group; P—pain group; GH—general health group).

## Data Availability

Data are available upon reasonable request.

## Supplementary Materials

The following supporting information can be downloaded at https://www.mdpi.com/article/10.3390/medicina61020292/s1, Figure S1: Random regression forest model architecture; Table S1: EQ-5D-5L ceiling effect, Shannon index (H’) and Shannon evenness index (J’) values; Table S2: Spearman correlations between different EQ-5D-5L questionnaire groups; Table S3: Random forest model results; Figure S2: Weighted frequency of absolute EQ-5D-5L overall score prediction errors in the random forest model for training (blue) and validation (red) data sets.
