# Supplementary material for: Assessment of Quality of Life in Lithuanian Patients with Multimorbidity Using the EQ-5D-5L Questionnaire
Source: Medicina (Kaunas). 2025 Feb 8;61(2):292. doi: 10.3390/medicina61020292 (PMC11857477; doi:10.3390/medicina61020292)
Supplement: Supplementary file 1 [file medicina-61-00292-s001.zip › medicina-3416543-supplementary.pdf]

medicina

MDPI

Supplementary Appendix

Links for the EuroQol EQ-5D-5L main website and registration website:

- https://euroqol.org/information-and-support/euroqol-instruments/eq-5d-5l/
- https://registration.euroqol.org/leads?regtype=2&regunsure=0

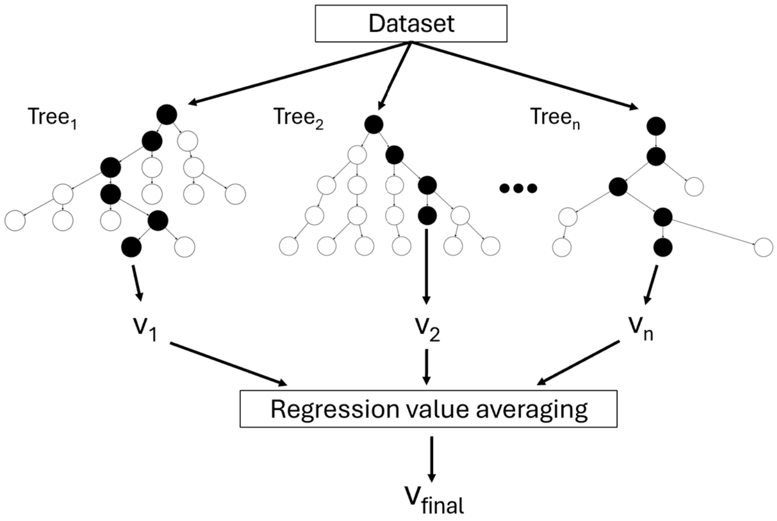

Supplementary Figure S1. Random regression forest model architecture. V – resulting values.

Supplementary Table S1. EQ-5D-5L ceiling effect, Shannon index (H') and Shannon evenness index (J') values.

	Ceiling effect (%)	H'	95% CI H' #	J'
Mobility*	48.795	1.697	1.696-1.699	0.731
Self-care	81.727	0.880	0.876-0.883	0.379
Usual activities	65.462	1.380	1.376-1.382	0.594
Pain/discomfort	25.301	1.838	1.837-1.838	0.791
Anxiety/depression	57.631	1.507	1.504-1.509	0.649
Overall**	16.000			

*The ceiling effect was calculated as the percentage of study participants who selected the "1 – no problems" option for the specific questionnaire categories.

CI – confidence interval calculated based on the Shannon index variance formula (citata Mlynczak)

** The overall ceiling effect was calculated as the percentage of study participants who selected the "1 – no problems" option for all of the questionnaire categories.

Supplementary Table S2. Spearman correlations between different EQ-5D-5L questionnaire groups.

	Mobility	Self-care	Usual activities	Pain/discomfort	Anxiety/depression
Mobility		0.433	0.538	0.526	0.180
Self-care			0.563	0.378	0.177
Usual activities				0.402	0.225
Pain/ discomfort					0.248

Supplementary Table S3. Random forest model results.

	RMSE value*	95th percentile of the training set RMSE	Percentage of RMSE values in the validation set that are below the 95th percentile of training data distribution (%)
Training set[a]	0.0565	0.116	65.71
Validation set	0.1396		

*RMSE – root mean square error

[a] The training and validation sets respectively had 70 and 30 percent of all RMSE data points. The error for each of the data points was considered to be the distance between the true EQ-5D-5L value for a data point and the prediction (e.g. generating a prediction of 0.73 for a data point that has a true value of 0.80 gives an error value of 0.07).

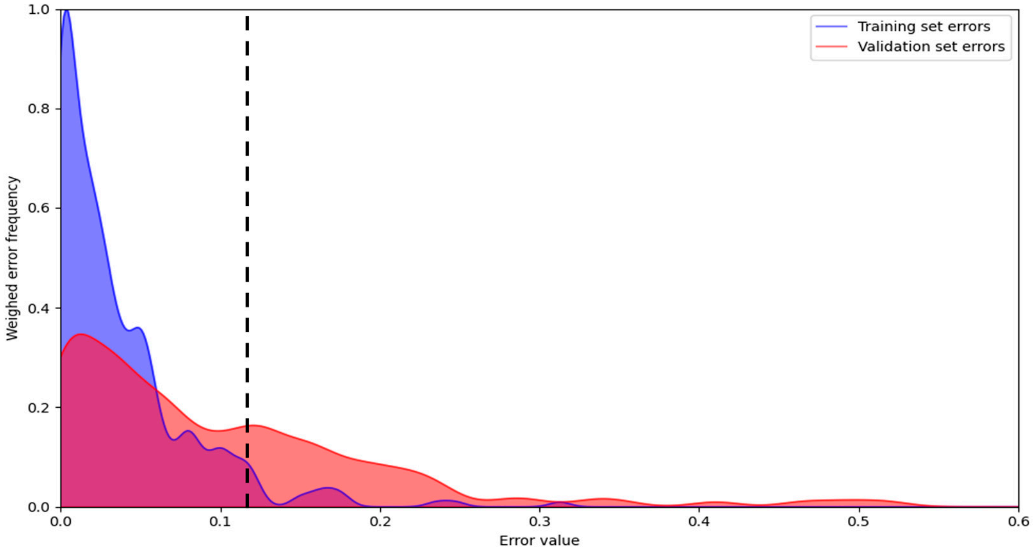

Supplementary Figure S2. Weighted frequency of absolute EQ-5D-5L overall score prediction errors in the random forest model for training (blue) and validation (red) data sets. The dashed line indicates the 95th percentile of the training dataset RMSE distribution (0.116) which is larger than 65.71% of the values in the validation set.
